# Estimation of cardiac output and systemic vascular resistance using a multivariate regression model with features selected from the finger photoplethysmogram and routine cardiovascular measurements

**DOI:** 10.1186/1475-925X-12-19

**Published:** 2013-03-04

**Authors:** Qim Y Lee, Stephen J Redmond, Gregory SH Chan, Paul M Middleton, Elizabeth Steel, Philip Malouf, Cristopher Critoph, Gordon Flynn, Emma O’Lone, Nigel H Lovell

**Affiliations:** 1 School of Electrical Engineering and Telecommunications, University of New South Wales, Sydney, NSW, 2052, Australia; 2 Graduate School of Biomedical Engineering, University of New South Wales, Sydney, NSW, 2052, Australia; 3 Australian Resusitation Council, NSW Branch, Brookvale, NSW, 2100, Australia; 4 Intensive Care Unit, Prince of Wales Hospital, Sydney, NSW, 2031, Australia; 5 Discipline of Emergency Medicine, University of Sydney, NSW, 2006, Australia

**Keywords:** Cardiac output, Systemic vascular resistance, Photoplethysmography, Power spectrum analysis, Photoplethysmogram variability, Photoplethysmogram morphology, Feature selection

## Abstract

**Background:**

Cardiac output (CO) and systemic vascular resistance (SVR) are two important parameters of the cardiovascular system. The ability to measure these parameters continuously and noninvasively may assist in diagnosing and monitoring patients with suspected cardiovascular diseases, or other critical illnesses. In this study, a method is proposed to estimate both the CO and SVR of a heterogeneous cohort of intensive care unit patients (*N*=48).

**Methods:**

Spectral and morphological features were extracted from the finger photoplethysmogram, and added to heart rate and mean arterial pressure as input features to a multivariate regression model to estimate CO and SVR. A stepwise feature search algorithm was employed to select statistically significant features. Leave-one-out cross validation was used to assess the generalized model performance. The degree of agreement between the estimation method and the gold standard was assessed using Bland-Altman analysis.

**Results:**

The Bland-Altman bias ±precision (1.96 times standard deviation) for CO was -0.01 ±2.70 L min^-1^ when only photoplethysmogram (PPG) features were used, and for SVR was -0.87 ±412 dyn.s.cm^-5^ when only one PPG variability feature was used.

**Conclusions:**

These promising results indicate the feasibility of using the method described as a non-invasive preliminary diagnostic tool in supervised or unsupervised clinical settings.

## Background

The volume of blood ejected by the heart per unit time is a vital physiological parameter known as the cardiac output (CO). CO varies, depending on the oxygen and nutrient requirements of the organs and tissues; the other major determinants of CO include blood volume status, cardiac contractility and systemic vascular resistance (SVR).

SVR is the aggregate resistance to blood flow in the systemic circulation. The amount of resistance to flow is related to the size of the blood vessel by Poiseuille’s law and vessel diameters are controlled by the sympathetic nervous system through variation of vasoconstrictor tone. Vessel sizes decrease and the SVR increases when there is an enhancement in this tone, while the suppression of this tone results in the opposite. An increase in resistance can lead to a decrease in CO, and vice versa.

Measurements of SVR and CO can provide important information about the overall hemodynamic performance of patients for diagnostic purposes. For instance, CO can be used to examine the cardiac status of critically ill patients and assist in the diagnosis of those with suspected cardiovascular diseases, like acute coronary syndromes, hypovolemia, valvular stenosis, myocarditis, cardiomyopathy and arteriosclerosis [1]. Low CO can indicate potentially adverse cardiovascular events such as cardiogenic shock [2]. Similarly, SVR can be a useful diagnostic tool, as deviation beyond the normal SVR range may be an indicator of critical illness. For example, an increase in SVR may be observed in hemorrhagic shock due to trauma, and ischemic and hemorrhagic stroke patients [3], while a depression in SVR is evident in distributive shock patients such as those with sepsis [4] or anaphylaxis. Continuous monitoring of SVR has been suggested as a diagnostic and research tool [5].

The current gold standard for CO measurement, the thermodilution technique [6], is an invasive procedure requiring the insertion of a pulmonary artery catheter. Recent developments have enabled CO to be estimated non-invasively (or with minimal invasiveness) using Doppler ultrasound, thoracic bioimpedance and pulse contour analysis [6-10] but few of these methods have not been used extensively in clinical settings [11]. Some of the reasons for the underutilization of these non-invasive methods include the requirement of a trained operator, the cost of the required specialized equipment and their disposable components, as well as the accuracy, precision and reproducibility of the measurement methods. As an example, the Doppler ultrasound method requires well-trained personnel to operate [12] and the uncertainty of the flow profile and diameter of the blood vessel contributes to the inaccuracy of the method [8,13] and the bioimpedance method has demonstrated poor results in numerous validation studies in critically ill and septic patients [8]. The minimally invasive pulse contour analysis technique requires continuous measurement of the arterial pulse pressure waveform from a peripheral artery (where the waveform itself can be measured invasively or non-invasively) as well as calibration against a standard method (such as transpulmonary thermodilution). Pulse contour analysis is considered minimally invasive because the patients in a critical care setting are assumed to already have the central venous and arterial cannulation required for transpulmonary thermodilution and thus no additional catheter is required [6]. The Finapres or the Portapres device [7,10], used with the Modelflow pulse wave analysis method, can be used to continuously monitor cardiac output non-invasively, but these devices are currently not widely used in the clinical setting. None of the existing non-invasive measurement methods are without drawbacks, or are suitable for all cohorts of patients, and the search for a low-cost, easy to use technique for different situations continues.

The photoplethysmogram (PPG) sensor is a non-invasive, low-cost and easy to use device that is routinely used in clinical settings to measure blood oxygen saturation levels. The device usually consists of light emitter (single or dual wavelength) and a photodetector, packaged in a small and highly portable form factor. The sensor is usually applied to the earlobe or the finger of the patient and it is comfortable enough to enable continuous measurement over a long period of time; in this study, the PPG signal was used as one of the inputs to estimate CO or SVR. The use of the PPG signal in the estimation of CO or SVR has been studied previously. Recently, Wang et al. proposed a method to estimate CO noninvasively using the PPG and electrocardiogram (ECG) during exercise that required an initial calibration [14]. In the studies by Awad et al., multi-linear regression analysis was performed to estimate CO and SVR from PPG features, such as pulse width (PW) [4,15]. Although the bias of their CO and SVR estimations was small, the precision was considered not sufficiently high for providing absolute values suitable for clinical use. A multivariate approach to classify SVR into discrete categories based on PPG features was previously developed by our research group and showed good results, especially in identifying patients with low SVR [16]; but the method did not provide an estimate of the actual SVR value. In many small hospital or prehospital care settings, the ability to follow the longitudinal trend of CO and SVR is very important as it enables monitoring of the effects of treatment on patient outcomes. The development of an approach that can provide an accurate estimate of SVR based on PPG measurement was therefore considered desirable, as it is the first step to enable trend monitoring.

In this study, it is proposed that a more accurate estimation of SVR and CO may be obtained using a multivariate regression model based on the use of PPG and routine cardiovascular measurements. Specifically, unique features extracted from the PPG variability (PPGV), which were anticipated to improve the estimation accuracy, were used in the regression model. PPGV is the beat-to-beat fluctuation in the PPG waveform and the spectral power distribution of the PPGV has been found to be correlated with SVR [17] and to be potentially useful for clinical diagnosis (e.g., sepsis [18,19]). In addition to the PPGV features, the multivariate model in this study incorporated pulse wave morphological features from the PPG waveform (the pulse width) to help estimate CO and SVR. Furthermore, stringent model selection and assessment of generalized performance of this estimation model were implemented with a nested leave-one-out cross validation procedure (LOOCV).

## Methods

### Database

The analysis was performed on data obtained from a heterogeneous group (*N*=48) of post cardiac surgery patients from the intensive care unit (ICU) at the Prince of Wales Hospital, Sydney, Australia, as described previously in [17]. The patients had the following physiological characteristics (mean ±SEM): 33 males and 15 females, age (69 ±1.5 yr), heart rate (HR, 84 ±2 bpm), CO (5.7 ±0.2 L/min), mean arterial pressure (MAP, 78 ±3 mmHg), central venous pressure (CVP, 14.5 ±0.7 mmHg) and SVR (926 ±36 dyn.s.cm^-5^). Thirty-six (*N*=36) of these patients were mechanically ventilated with a respiratory rate of approximately 9-15 breaths per minute and a tidal volume of approximately 500 mL. Written informed consent was acquired from each patient or his/her next of kin prior to inclusion in the study. The study was approved by the Human Research Ethics Committee of the Prince of Wales Hospital, and conducted according to the Australian national guidelines on ethical research involving human subjects, as well as the World Medical Association Declaration of Helsinki.

Using a Universal Clinical Workstation Monitor (Spacelabs Healthcare, WA, USA), HR, CO, systolic and diastolic blood pressures and CVP were measured from each patient in the supine position. Arterial blood pressure was measured invasively using a radial artery catheter, and MAP was subsequently recorded. CVP was measured invasively using a catheter inserted into the superior vena cava while the CO was measured via a pulmonary artery catheter using the thermodilution method. No other interventions were performed during the measurement period. SVR, in units of dyn.s.cm^-5^, was calculated from other measurements using the following formula:

(1) $$\text{SVR}=\frac{80\times (\text{MAP}-\text{CVP})}{\text{CO}}.$$

Following the measurement of CO, the PPG signal was recorded from the tip of the right index finger of each patient using a reflection mode infrared (940 nm) finger probe (ADInstruments, Sydney, Australia) connected to a PowerLab data acquisition system (ADInstruments, Sydney, Australia). The PPG signal was sampled at either 200 Hz or 1 kHz and recorded for a duration of approximately 10 min. The signal was not subjected to any high-pass filtering prior to sampling to preserve the baseline variation. As described in a previous publication [17], the 48 sets of data used in the analysis were obtained after the exclusion of 16 sets of poor quality PPG signals (from a larger pool of 64 subjects) which contained severe motion artifact or baseline drift, frequent ectopic (abnormal) beats, and barely recognizable cardiac pulses which prohibited reliable pulse detection.

### PPG signal analysis and feature extraction

The derivation of various PPG features and their respective meanings have been explained in detail in a previous publication by the authors [16]. The two main categories are the spectral features derived from the low frequency (LF, 0.04-0.145 Hz), mid frequency (MF, 0.08-0.145 Hz) and high frequency (HF, 0.145-0.45 Hz) bands and the morphological feature. Spectral features include the normalized low-frequency power (*LF*_*NU*_), the normalized mid-frequency power (*MF*_*NU*_) and the low-frequency to high-frequency power ratio (*LF*/*HF*) of the PPGV. The morphological feature used is the pulse width (*PW*), which is the normalized width (in time) of the PPG pulse at half of its amplitude (peak to trough). In addition to the PPG signal features, the additional non-PPG-based features of HR, MAP and the ratio of MAP to HR (*MAP*/*HR*) were also used. In summary, there are three PPGV (spectral) features, one PPG morphological feature and three non-PPG-based features, giving seven features in total. Several notch-related features described in the previous work of the authors [16] were not included in the feature pool because not all patients had a clear dicrotic notch or inflection point. This complicates the accurate extraction of the feature value. It was found that these features caused unstable LOOCV feature selection (described in a later subsection) when left in the feature pool.

### Regression analysis

The CO or SVR was estimated as a weighted sum of a chosen subset of *M* features from the pool of all extracted features, described in the previous section, by means of linear least square modelling. In the following exposition, the unknown variable to be estimated is assumed to be CO, but the methods are equally applicable to estimate SVR.

For each CO, denoted *y*_*i*_,*i*=1,2,…,*N*, assumed to be related to the selected features ${\tilde{x}}_{i}={\left[x_{i1},x_{i2},\ldots ,x_{\text{iM}}\right]}^{T}$ by a set of weights $\tilde{w}={\left[w_{1},w_{2},\ldots ,w_{M}\right]}^{T}$ and a constant *w*_*M*+1_ as follow

(2) $${ŷ}_{i}={\tilde{x}}_{i}^{T}\tilde{w}+w_{M+1}.$$

In matrix form,

(3) $$\begin{array}{l} \hat{y}={\tilde{X}}^{T}\tilde{w}+w_{M+1}\cdot 1\\ =[{\tilde{X}}^{T}|1]w\\ =Xw \end{array}$$

where $\hat{y}$ is a column vector and the *j*-th row of $\hat{y}$, ${ŷ}_{i}$, is the CO of the *j*-th subject; **X** is a matrix with the *j*-th row representing the *M* features of the *j*-th subject augmented with a column of ones, **1**; and **w**=[*w*_1_,*w*_2_,…,*w*_*M*_,*w*_*M*+1_]^*T*^.

The set of weights, **w**, which defines the CO least square model for the *M* selected features, can be solved by using the relationship

(4) $$w=X^{+}\hat{y}$$

where **X**^+^ is the Moore-Penrose pseudoinverse of the matrix **X**.

An unknown CO value can be estimated using the set of features extracted from the PPG waveform from the relationship in (2), once the appropriate weights have been discovered. The mean-squared error (*MSE*) between the measured and the estimated CO (or SVR) of the model is calculated as

(5) $$\text{MSE}=\frac{1}{48}\overset{48}{\underset{i=1}{\sum }}{\left({ŷ}_{i}-y_{i}\right)}^{2},$$

where *y*_*i*_ is the gold standard measure of CO for the *i*-th subject. The *MSE* may be used as a measure of how well the estimated CO agrees with the measured gold standard CO. The set of features were selected using a feature selection algorithm and the model is then validated using the LOOCV method, as described in the following section. The degree of agreement between the estimated and the measured CO is evaluated using Bland-Altman plots [20].

### Feature transformation

The squared and cubed value, as well as the logarithms of each feature is added into the feature pool to expand the seven existing features to a total of 28 features in the feature pool. These features, referred to as the transformed features in this paper, are included to ensure any non-linear relationship between the features and the CO is captured by the multivariate model.

### Leave-one-out cross validation (LOOCV)

If all of the subjects are used in the training phase to obtain the model (weight vector **w**) which is subsequently used to estimate *y*_*i*_, the *MSE* in (5) will typically be underestimated. This is due to the reason that the model is optimized to reduce the *MSE* of the training data, but not on unseen data. Models that do not generalize well to new data are said to be “overfitted” to the training data. The data used for testing must not be used in either the training or feature selection phases in order to obtain an unbiased measure of the generalized performance (expected error rate for new, unseen data) when testing is done.

In this study, a nested leave-one-out cross-validation scheme was used. This was achieved by repeatedly dividing the data into three sets (training, feature selection and test sets) such that the data in the test set did not appear in the feature selection or training sets for a given iteration.

**Figure 1:**
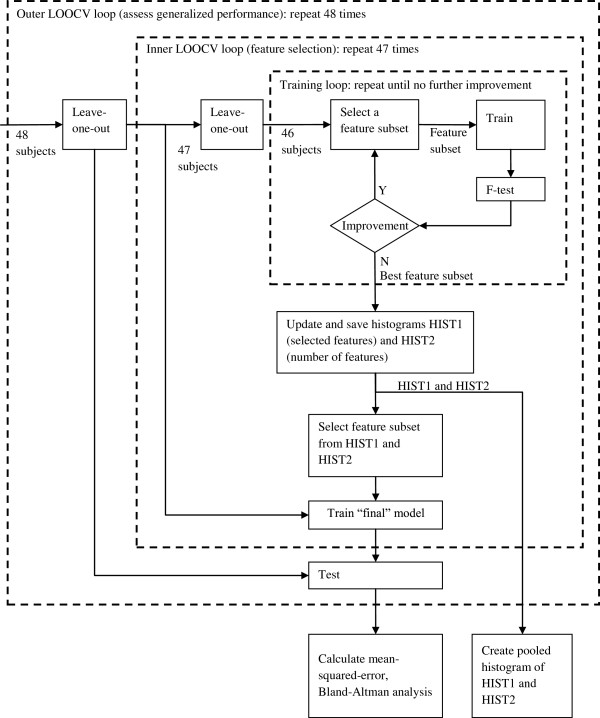
**Block diagram of the nested LOOCV procedure.** In the inner most loop, the search for the best feature subset (for the 46 subjects in that loop) is performed by sequentially adding/removing features into/from the model, followed by an F-test to check the improvement made to the model. The middle loop is repeated 47 times, where each repetition returns a “final” model, as determined by HIST1 and HIST2, to the outer most loop. In the outer most loop, an LOOCV is performed where a test subject data was withheld and used to test the model returned by the middle loop, which has no prior information on the withheld data. The generalized performance was estimated from the 48 test results, and pooled histograms of HIST 1 and HIST2 were plotted to determine which features were most useful in estimating CO and SVR.

Figure 1 shows the block diagram of the nested LOOCV. The procedure used to implement the nested LOOCV was as follows. In the outer LOOCV loop, one set of subject data was set aside to be used as the test data. The remaining subjects’ data (N=47 in our case) was used in the inner LOOCV loop to select features and train a regression model to be tested on this withheld subject. This procedure was repeated 48 times, leaving out one subject on each run.

In the inner loop, another LOOCV procedure was used to select a set of features. One set of subject data was removed and the remaining 46 subjects’ data was used to select a set of features, based on a statistical F-test of the significance of their inclusion in the multiple regression model; the details of this procedure are outlined in the next section. To improve the reliability of the features selected, This inner loop was repeated 47 times by sequentially setting aside one set of subject data from the set of 47 subject data available. Histograms of the frequency of a certain feature being selected across these 47 runs (HIST1) and of the number of features selected in each run (HIST2) were used to select the final subsets of features employed in the model used to test the outer-most withheld (48th) subject against. The specifics of how HIST1 and HIST2 are used to select the final feature set for testing is described in the following example.

Suppose if after 47 repeats of feature selection, three features are most commonly selected according to HIST2, then, the three most frequently selected features from HIST1 should be used to build the model.

In the outer LOOCV loops, once the model size and features to be used were fixed, the model was retrained using all the 47 sets of subject data, and the one set that was left out earlier was used as the test set. This ensures that testing is performed on data unseen before in the training phase. The estimation error for each subject was calculated and the MSE calculated using (5).

### Feature selection

In the inner most loop of Figure 1, a stepwise feature selection was employed. The regression model is initiated with only a constant term, with each of the available features being sequentially added if they improve the model. The improvement to the current model was evaluated using the partial F-statistic on the sum of squared error [21]. In brief, the F-statistic quantified the amount of improvement due to adding a feature to the model, for a given sample size. If a feature was statistically significant (at 95% confidence level), it was added to the model. After every step of feature addition, the features that were already selected were tested to check if their removal would improve the model (also at 95% confidence level). The search stopped when no feature addition or removal resulted in a significant improvement in the model. The procedure described above was repeated 47 times, leaving one subject’s data out, so that the results of these 47 different searches can be pooled to create HIST 1 and HIST2.

The entire procedure described in Figure 1 was performed several times, using different starting feature pools. To study the usefulness of the PPG signal in estimating CO and SVR, features not directly derivable from the PPG signal (such as MAP) were removed from the feature pool when the feature selection algorithm was executed. Furthermore, to study the usefulness of PPGV features in the estimation task, a feature pool that contained only the PPGV features was used. The performances of the regression models selected in these two cases were compared to the model found from the complete feature pool, and comparisons of performances between models that used only a single feature (univariate models), where feature selection becomes trivial, were also made.

### Bland-Altman analysis

Agreement between the methods in this study was calculated using the bias and precision plot method described by Bland and Altman [20,22-24].

In addition to the correlation, bias and precision performance, the percentage error of the estimation method, calculated as 100×precision divided by mean (average of all subject CO or SVR values), was also reported to compare the agreement of the estimation method with the gold standard.

## Results

Table 1 lists all the features in the feature pool and their associated number, which are used to reference the feature in the discussions that follows. Table 2 shows the performance figures of the regression model for CO and SVR estimation, for each of the different starting feature subsets drawn from the entire feature pool. The correlation, bias (mean difference between measured and estimated value) and the precision (1.96 ×standard deviation (s.d.) of the differences) of the estimation method when compared to the thermodilution method, calculated using the Bland-Altman analysis, and the percentage error are also shown in Table 2. Note that the correlation value is the correlation of the measured value (CO or SVR) with the estimated output which was subjected to nested LOOCV.

**Table 1:** Feature pool and their respective reference numbers

**Feature**	***x***	***x***^**2**^	***x***^**3**^	**log(*x*)**
*LF*_*NU*_	1	8	15	22
*MF*_*NU*_	2	9	16	23
*LF*/*HF*	3	10	17	24
*HR*	4	11	18	25
*MAP*	5	12	29	26
*MAP*/*HR*	6	13	20	27
*PW*	7	14	21	28

Each number represents a feature (left headings) and the transformation (top headings) that is used. For example, feature 1, 14 and 25 denotes *LF*_*NU*_, *PW*^2^ and log(*HR*), respectively.

**Table 2:** CO and SVR estimation performance and selected features

**Estimated variable**	**r**	**Bias**	**s.d.**	**% error**	**Feature pool**
CO	0.45	0	1.49	51%	All features
(L min^-1^)	0.54	-0.01	1.38	47%	Only PPG features (i.e., all except 4-6, 11-13, 18-20,25-27)
	0.42	0	1.49	51%	Only PW
	0.39	-0.01	1.51	52%	Exclude PPGV (i.e., all except 1-3, 8-10, 15-17, 22-24)
	0.30	-0.01	1.62	55%	Only linear features (exclude 8-28)
SVR	0.50	1.45	236	50%	All features
(dyn.s.cm^-5^)	0.55	6.50	211	45%	Only PPG features (i.e., all except 4-6, 11-13, 18-20, 25-27)
	0.14	-6.30	256	54%	Only PW
	0.40	3.17	233	49%	Exclude PPGV (i.e., all except 1-3, 8-10, 15-17, 22-24)
	0.52	-7.28	217	46%	Only linear features (exclude 8-28)

The performance figures achieved by the feature selection algorithm to estimate CO and SVR using different starting feature subsets drawn from the entire feature pool. The correlation, Bland-Altman bias and s.d., as well as the percentage error achieved is shown.

**Figure 2:**
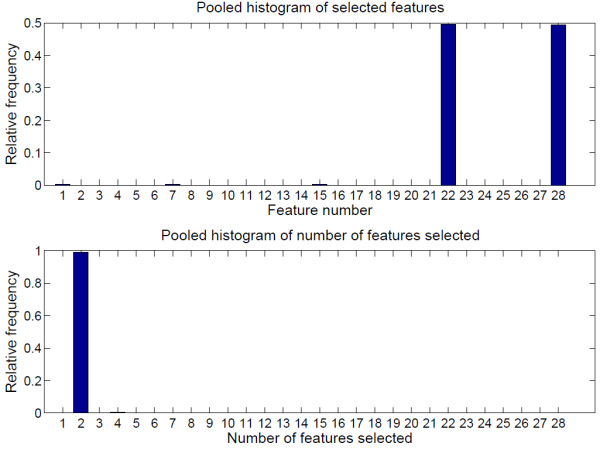
**Pooled histogram of selected features and number of features used when estimating CO.** Top: Pooled HIST1 - relative frequency of a feature being selected to estimate CO, bottom: Pooled HIST2 - relative frequency of number of features being used in the CO estimation model. Two features were most commonly selected by the feature selection algorithm; feature 22 (log(*LF*_*NU*_)) and feature 28 (log(*PW*)).

**Figure 3:**
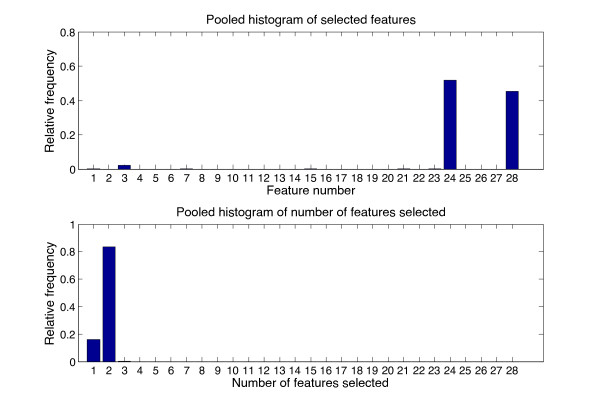
**Pooled histogram of selected features and number of features used when estimating SVR using the best multivariate model.** Top: Pooled HIST1 - relative frequency of a feature being selected to estimate SVR, bottom: Pooled HIST2 - relative frequency of number of features being used in the SVR estimation model. Two features were most commonly selected by the feature selection algorithm; feature 24 (log(*LF*/*HF*)) and feature 28 (log(*PW*)). Feature 3 (LF/HF) was sometimes selected.

Figure 2 is the pooled histogram of the selected features used to estimate CO (top) and the pooled histogram of the number of features used to estimate CO (bottom). In other words, Figure 2 is the sum of the 48 HIST1 and HIST2, where each of them is a histogram generated by leaving out one subject before feature selection proceeds. These histograms were pooled to study which features were most influential, and how many features were usually selected. Note that the features were not selected based on this figure because it contains information on all subjects including the test subject and thus, will result in a biased estimate of the performance. Figure 3 is similar to Figure 2, with the estimated variable being SVR.

**Figure 4:**
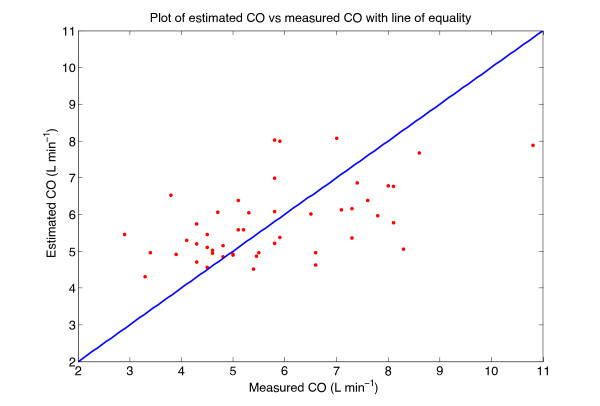
**Plot of estimated CO against measured CO.** Plot of estimated CO against measured CO for all 48 subjects, with the line of equality.

**Figure 5:**
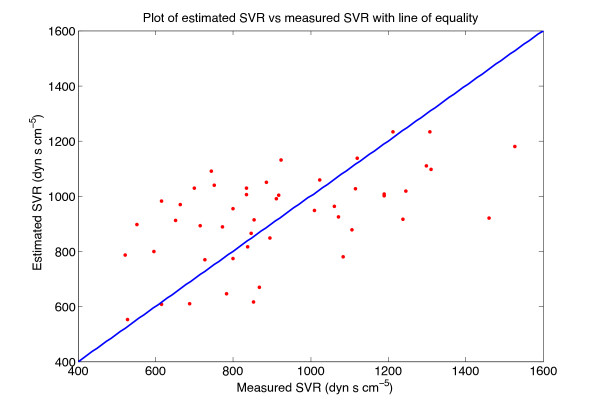
**Plot of estimated SVR against measured SVR.** Plot of estimated SVR against measured SVR for all 48 subjects, with the line of equality.

**Figure 6:**
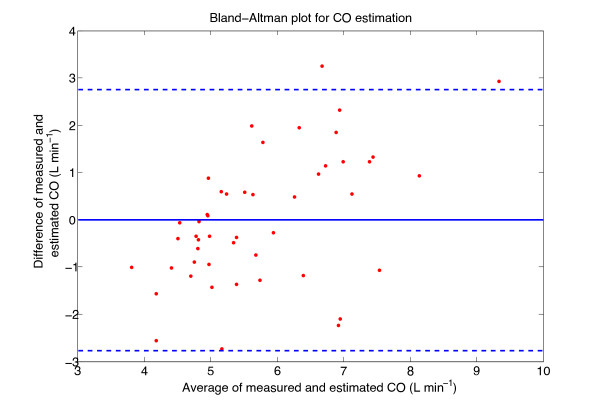
**Bland-Altman plot of the CO estimation.** The solid line in the middle is the bias and the two lines above and below are the limits of agreement, calculated as bias ±1.96×s.d.. Bias = -0.01 L min−1, 1.96×s.d. = 2.70 L min−1.

**Figure 7:**
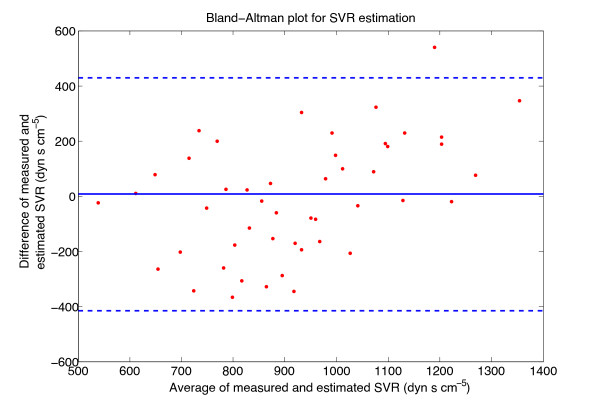
**Bland-Altman plot of the SVR estimation.** The solid line in the middle is the bias and the two lines above and below are the limits of agreement, calculated as bias ±1.96×s.d.. Bias = -0.87 dyn.s.cm^-5^, 1.96×s.d. = 412 dyn.s.cm^-5^.

Figures 4 and 5 show plots of the estimated variable plotted against the measured variable, along with the line of equality, for the best CO and SVR models, respectively. The Bland-Altman plot of the best CO and SVR estimations are depicted in Figures 6 and 7.

The performance indicators (correlation, bias and s.d.) for models constructed using a single feature are shown in Table 3 for comparison with the multivariate model, for both CO and SVR. Due to space constraints, only the ten best single features are shown in the table, in descending order of s.d.. The correlation shown is the correlation of CO or SVR with the estimated output subjected to LOOCV, not the correlation of the feature itself with the CO or SVR.

**Table 3:** CO and SVR estimation performance of single feature models

**Feature**	**CO (L min−1)**			**Feature**	**SVR (dyn.s.cm**^**-5**^**)**		
	**r**	**Bias**	**s.d.**		**r**	**Bias**	**s.d.**
28	0.42	0	1.49	24	0.55	-0.87	210
7	0.41	-0.01	1.50	15	0.52	-0.45	215
14	0.38	-0.01	1.52	8	0.52	-0.47	215
21	0.34	-0.01	1.55	1	0.51	-0.57	216
24	0.32	0	1.56	3	0.51	1.85	218
22	0.32	0	1.56	22	0.46	-1.13	224
25	0.28	0.01	1.58	13	0.45	0.13	225
1	0.28	0	1.58	6	0.44	-0.46	226
4	0.25	0.01	1.59	20	0.45	0.92	226
23	0.24	0	1.60	27	0.43	-0.89	228

The Bland-Altman bias and s.d. achieved when a univariate model is used. Only the 10 best single features are shown here, in increasing order of s.d.. The best s.d. achieved when estimating CO using a multivariate model constructed using only PPG features was 1.38 L min^-1^. For SVR, the multivariate model has an s.d. of 211 dyn.s.cm^-5^.

## Discussion

In this study, multivariate regression models were developed to estimate CO and SVR using a combination of features extracted from the PPG waveform, other routine cardiovascular measurements and the non-linear transformations of these features (quadratic and cubic powers and logarithm transform). The bias ±precision (1.96 ×s.d.) for the best CO model found was -0.01 ± 2.70 L min^-1^, and for the SVR model, the best bias precision achieved was -0.87 ±412 dyn.s.cm^-5^. Note that these numbers, and those in the discussions that follow, are stated as bias ±1.96×s.d..

### CO estimation

The relationship between CO and features extracted from the finger and ear PPG was previously investigated by Awad *et al*. It was found that both finger and ear PPG widths have significant correlation with the CO, the reason being an increase in CO increases the amplitude but decreases the duration of the PPG waveform [15]. However, their multi-linear regression model did not provide an estimate with good precision (s.d.=2.46 L min−1). The authors suggested that non-linear analysis or application of a calibration technique may be useful to improve the estimate. In this study, PW was found to have mild but significant relationships with CO (*r*=−0.48,*p*<0.0001; note that this is a direct correlation coefficient with CO not subjected to LOOCV). While it is difficult to compare the estimation precision achieved in this study to that of the study by Awad *et al.*, given the difference in clinical setting, subject cohort, signal/features used (finger instead of ear PPG pulse width) and the range of CO, it was found in this study that the utility of non-linear transformation on the features slightly improved the CO estimation performance from -0.01 ±3.18 L min−1 to -0.01 ±2.96 L min−1, when PPGV features (*LF*_*NU*_, *MF*_*NU*_ and *LF*/*HF*) were not used. When all features are included in the pool, the performance increased slightly to 0 ±2.92 L min−1.

Further analysis revealed that excluding MAP and HR related features, i.e., using only PPG features, further improves the CO estimation bias and precision (-0.01 ±2.70 L min^-1^). This means that a reasonable estimate of CO can be obtained using only the PPG waveform. From Figure 1, it can be seen that log(*LF*_*NU*_) and log(*PW*) were consistently selected to estimate CO. *LF*_*NU*_ has been found to be related to the degree of vasodilatation [17], and thus, may be used as a predictor variable for CO. *PW* was also found to be correlated to CO in another study [15].

The percentage error of the estimation was 47%. Assuming that the thermodilution method has up to 20% measurement error, the error rate was above the 30% error threshold for the acceptance of a new method, as proposed by Critchley and Critchley [25]. The performance achieved here does not reach this threshold. However, it should be noted that the above assumption regarding the acceptable percentage error of the thermodilution method is still a matter of debate [11,26], and so, the error threshold could be higher than 30% for a new method to be accepted. Although it cannot be used interchangeably with the ’gold standard’ at this stage, given the portability and ease of use, the method proposed here may nevertheless prove to be a viable alternative in unsupervised clinical or emergency triage settings, in which other alternative methods may not be practically applied.

### SVR estimation

The research literature describing the measurement of SVR using non-invasive means is not as extensive as the volume of publications on the same topic of CO, but this does not lessen the importance of this hemodynamic parameter. Trends in SVR index were monitored using invasive methods during different clinical interventions, in a study by O’Dwyer *et al.*[5], and it was concluded in that study that continuous monitoring of the SVR has much potential as a diagnostic and research tool, for example, in chest physiotherapy studies, and as a rapid accurate assessment of patient response to therapy, such as the effects of infusion of vasoactive drugs.

In a previous study from our research group, the *LF*_*NU*_ extracted from the PPGV was found to be useful for identifying patients with a low SVR (<900 dyn.s.cm^-5^) with a high specificity [17]. The LF of the PPGV was used because it was found that an increase in LF power was linked with sympathetic activation. The work was further developed to categorise patients into different levels of SVR using a multivariate classifier [16]. Awad *et al.* studied the viability of estimating SVR using a linear multiple regression model and features from the PPG waveform, namely, the ear and finger PPG pulse width and ear PPG pulse area [4]. It was found that the ear PPG has significant correlation with the SVR, and the authors explained that it was a result of a prolonged transit time of the blood flow due to peripheral vasoconstrictions. Although the estimation bias achieved in that study was small (29.8 dyn.s.cm^-5^), the limits of agreement were admittedly too large (s.d.=587.3 dyn.s.cm^-5^).

In the analysis presented here, a quantitative estimate of the SVR with bias ±precision of 1.45 ±463 dyn.s.cm^-5^ was achieved using a multivariate regression model constructed from PPG features and routine cardiovascular features. If PPG features alone were used in the model, the bias ±precision performance improved to 6.5 ±414 dyn.s.cm^-5^. The spectral feature log(*LF*/*HF*) was consistently selected to estimate SVR, while the feature log(*PW*) was selected 45 out of 48 times. The univariate model provided better estimation of the SVR (−0.87±412 dyn.s.cm^-5^) than the multivariate model, using only feature 24 (log(*LF*/*HF*)). Also note from Table 2, that a regression model using the PPGV feature log(*LF*/*HF*) provided better estimation than a regression model using only PW features (−6.3±502 dyn.s.cm^-5^).

### Limitations

One of the major limitations in this study is the PPG signal quality degradation caused by movement artifact, baseline drift, frequent ectopic beats or poor peripheral perfusion leading to weak and unrecognizable cardiac pulses, which resulted in the exclusion of sixteen sets of patient data. It is hoped that the promising results from this study and other PPG applications related studies will motivate improvement in PPG sensor technology and feature extraction algorithms in the future to mitigate these problems. One example of such improvement involves the integration of an accelerometer motion sensor to reject movement artifact [27].

The small sample size posed another challenge in developing the multivariate model and assessing the validity of the model developed. Ideally, the model should be trained using as many samples as possible to reflect the true population, but the model should be tested using samples not used in the training phase. This problem was addressed by using a nested LOOCV for both feature selection and testing to obtain a fair estimate of the generalized model performance. A larger training set is therefore desirable to definitively demonstrate the performance observed in this pilot study with a reduced chance of Type-I error.

## Conclusion

This study provides a preliminary indication of the potential usefulness of a method to non-invasively estimate CO and SVR using PPG signal. For the estimation of both CO and SVR, it was shown that the incorporation of spectral features complements and improves estimation achieved over using PW alone. The promising results obtained encourage further research to validate the method in a larger cohort, ultimately enabling a non-invasive, low-cost and easy to deploy alternative for estimating or tracking CO and SVR in clinical triage or unsupervised clinical settings, where the observation of trends in CO and SVR would have great value.

## Competing interests

The authors declare that they have no competing interests.

## Authors’ contributions

QYL, SJR, GSHC and PMM proposed the idea. GSHC, ES, PM, CC, GF and EO carried out the experiment and data collection. QYL, SJR and GSHC designed and implemented the algorithm and data analysis. QYL drafted the manuscript. QYL, SJR, GSHC, and NHL provided critical revision of the manuscript. SJR, GSHC, PMM and NHL supervised the study. All authors read and approved the final manuscript.
